# Integrative taxonomy uncovers a new stygobiotic *Caridina* species (Decapoda, Caridea, Atyidae) from Guizhou Province, China

**DOI:** 10.3897/zookeys.1028.63822

**Published:** 2021-04-05

**Authors:** Shuo Feng, Qing-Hua Chen, Zhao-Liang Guo

**Affiliations:** 1 Department of Animal Science, School of Life Science and Engineering, Foshan University, Nanhai 528231, Guangdong Province, China Foshan University Nanhai China; 2 South China Institute of Environmental Sciences, Ministry of Ecology and Environment, Guangzhou 510520, Guangdong Province, China South China Institute of Environmental Sciences, Ministry of Ecology and Environment Guangzhou China

**Keywords:** *
Caridina
*, COI, conservation biodiversity, freshwater biodiversity, southwestern China, speleology, taxonomy

## Abstract

Collecting much-needed information on the taxonomy, distribution, and ecology of cave-dwelling shrimp is vital for addressing the urgent challenges in conservation biodiversity in fragile cave ecosystems. *Caridina incolor***sp. nov.**, a new atyid shrimp from an underground stream of Yaoshui Cave, Daqikong scenic area, Libo County, Guizhou Province, southwestern China is described based on morphology and DNA analysis (mitochondrial COI). *Caridina incolor***sp. nov.** differs from epigean congeners by its smaller eyes which range from reduced to completely blind; colorless body and appendages; long stylocerite and sixth abdominal segment; and relatively large eggs. In comparison to other cave species, *Caridina incolor***sp. nov.** presents a long rostrum and stylocerite; slender sixth abdominal segment; and unique shape of the appendix masculina. Data on the habitat, ecology, and levels of threat are provided and suggest that it should be categorized as Critically Endangered (CR) under the current IUCN criteria.

## Introduction

China is rich in subterranean environments, with more than 500,000 documented caves, most of which are located in the southwest karst region, such as Guangxi, Guizhou, and Yunnan Province (Chen 2006; Ran and Yang 2015). This underground setting has a unique ecological habitat characterized by permanent darkness, relatively constant air and water temperature, and scarcity of food supply (Rétaux and Casane 2013; Culver and Pipan 2019; Mammola et al. 2019). They may have served as faunal refuges and indeed are known to harbor an impressive array of shrimp species (Holthuis 1974, 1977; Hobbs et al. 1977; Hart and Manning 1981; Liang and Yan 1981; Mejía-Ortíz and Hartnoll 2006; Li 2007; Cai and Ng 2009, 2018; Pan et al. 2010; Zhu et al. 2020). Though the stygobiont shrimps are prone to geographic and genetic isolation, they exhibit a suite of convergent characteristics under natural selection, including reduction or loss of eyes, loss of pigment, elongated antennas and ambulatory appendages. Non-visual sensory structures are enhanced with the loss of vision, which ultimately leads to speciation (Barr 1968; Holthuis 1986; Peck 1986; Christiansen 2005; Mejía-Ortíz et al. 2006). They serve an important underground ecological role and have a position near the base of the food chain. By consuming organic matter such as leaves and twigs that get flushed into caves, they are the primary decomposers and make nutrients available to other organisms in the ecosystem, such as fish and crabs (Botosaneanu 1985).

In China, most cave systems have not been adequately surveyed because of the difficulty in sample collection. To date, 24 described species of four genera of atyids are presently known as inhabitants of the subterranean aquatic realm, some of which are completely adapted to subterranean life, and the majority from the genus *Caridina* H. Milne Edwards, 1837 (Liang and Yan 1981; Guo et al. 1992, 1996; Liang and Zhou 1993; Cai 1995; Cai and Li 1997; Cai and Liang 1999; Cai and Ng 1999, 2018; Liang et al. 1999, 2005; Li and Li 2010; Xu et al. 2020).

Guizhou Province is the central area of karst landforms in southwest China, with 73% of the land covered with carbonate rocks (He and Li 2016; Zhou et al. 2017). The karstic nature of this area promotes the formation of submerged cave systems, with more than 700 large cave systems documented (Zhang and Zhu 2012). Although Guizhou presents great biospeological potential, most submerged cave systems have not been adequately surveyed or studied. The research concerning the cave-dwelling atyids in Guizhou started with a study by Cai and Li (1997), who described *Caridina demenica* Cai & Li, 1997 with pigmented reduced eyes from Libo County. Liang et al. (2005), subsequently revealed another three new atyids: *Caridina caverna* Liang, Chen & Li, 2005 which is blind and depigmented; *C. acuta* Liang, Chen & Li, 2005 which has slightly reduced eyes and pigmentation; and *Neocaridina brevidactyla* Liang, Chen & Li, 2005 which apparently does not show any adaptation to subterranean life. Cai and Ng (2018) reported a new cave-dwelling atyid *Caridina jiangkou* Cai & Ng, 2018 from Jiangkou County, which is also of the typical epigean form. More recently, Xu et al. (2020) reported *Caridina sinanensis* Xu, Li, Zheng & Guo, 2020 from Sinan County, which is unpigmented, and the cornea is vestigial with only a small pigment spot.

During our biospeleological surveys in Guizhou Province, stygobiont atyid shrimps belonging to the genus *Caridina* were collected from Yaoshui Cave, Daqikong scenic area, Libo County. The specimens collected could not be assigned to any known species of this genus based on a combination of morphological and molecular features (COI). *Caridina incolor* sp. nov. is rare and has a restricted distribution; its taxonomic uniqueness also suggests that it may be relictual. The impact of anthropogenic activities on the new species are also noted and suggests it is in need of urgent conservation intervention.

## Materials and methods

### Study cave and ecological data

Daqikong scenic area is named after a seven span bridge on the Dagou River near Mengtang Village, Wangmeng Township. It is situated about 25 km southwest of Libo County, at the border region of Guizhou and Guangxi in southwestern China. Yaoshui Cave is near the Mengtang scenic spot of Daqikong scenic area, at 25°17'1"N, 107°45'8"E and an altitude of 520 m. The entrance is about 120 m away from the tour plank road, located halfway up a limestone hill. The opening of the cave is arched and 2 m wide and 4 m high. There is naked shale above the entrance, with some ferns, bryophytes and vines present in the surrounding areas (Fig. 5A). Beyond the entrance is a rocky horizontal passage which is 2 m wide. Shrimps were observed in a pool (2 m^2^, 0.8 m deep, and in the weak light zone) situated 2 m from the entrance. The weak light zone channel is rugged and can only be entered along the tunnel wall. Another 4 m in is the underground river (in total darkness). In some areas, it is shallow (0.1–0.5 m) with a muddy bottom. However, in the deeper parts (1.3–2.5 m), the bottom becomes rocky and slopes precipitously to unknown depths. A number of shrimps were taken in the shallows. This cave was inhabited by bats and a thick layer of bat guano was also found on the ground.

**Figure 1.**
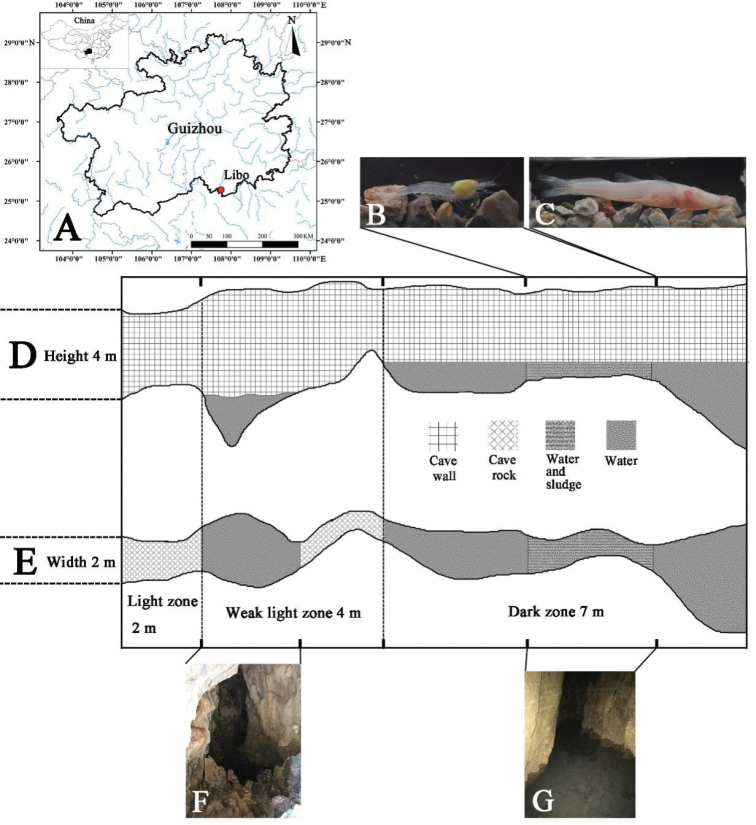
Type locality and schematic diagram of the cave of *Caridina incolor* sp. nov. **A** Yaoshui Cave is located near Libo County and is marked by the red dot **B** the sampling point of shrimp **C** the sampling point of fish **D** side view of the cave **E** plan view of the cave **F** a pool in a weak light zone **G** river in the dark zone.

During the November collection trip, the water was clear and the water parameters of the river were: temperature 21 °C, pH 7.5, and dissolved oxygen 8.8 mgl^-1^. Water levels of Yaoshui Cave fluctuate dramatically throughout the year. During spring and summer, heavy precipitation inundates the passage and blocks access to the entrance. Also, an outflowing stream snakes down into Dagou River (Fig. 5B). During autumn and winter, due to lower water levels, some pools may become dry, but the underground river does not dry up. The outline of the Yaoshui Cave is shown in Figure 1. The cave was visited by Deng et al. in 2011. Twenty-six cavefish specimens were collected and a new species, *Oreonectes daqikongensis* Deng, Wen, Xiao & Zhou, 2016 was described. They mention that only the blind fish was found in the underground river, and no other fish, shrimp, or aquatic animal were found (Deng et al. 2016). This blind fish was collected in the deeper parts of the river during this survey (Fig. 1C).

**Figure 2.**
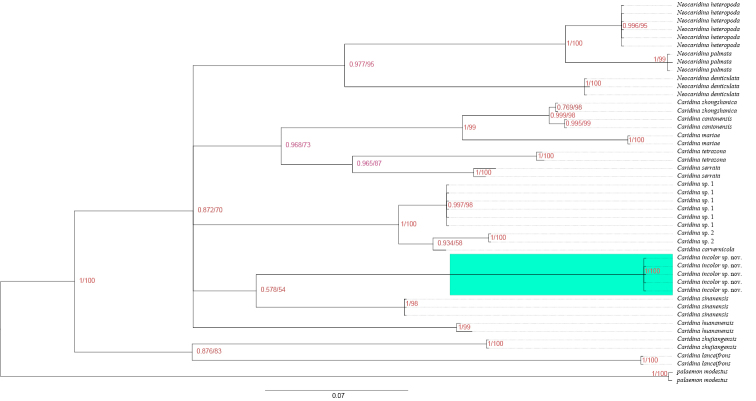
Bayesian inference (BI) tree and maximum likelihood method (ML) tree of 16 atyids and outgroups (*Palaemon modestus*) based on the COI gene. Support values at the nodes represent posterior probability.

### Sample collection

On August 3, the cave gallery was inundated due to the rain. Sample collectors managed to wade in, but only a juvenile female was collected. On November 27, the water level was lower, sample collectors successfully entered the cave. Under the light of our headlights, a school of translucent white shrimps were observed swimming or clinging to the cave wall of the river. Forty-two individuals were collected in the shallow muddy areas. Samples were collected with a hand net (mesh size 0.6 mm). The sampling scene was recorded with photographs and video-recordings. Specimens were placed in oxygenated polythene bags, anaesthetized with ice, then transported back to the hotel. The shrimps were fixed in 95% ethanol after they were photographed. The ethanol was changed after 24 h with fresh 75% ethanol. The blind fish (*Oreonectes daqikongensis*) was also collected. Basic hydrological and physicochemical parameters of the cave were measured by the following instruments: Bosch GLM-30 Laser rangefinder, eTrex Venture GPS locator, JWSA2-2 temperature and hygrometer, BDO820 Portable dissolved oxygen determining meter, and BPH-220 pH measuring apparatus.

### Morphological analysis

Specimens were examined using a dissecting microscope (Olympus SZX7). Morphometric measurements on selected characters and illustrations were made using a digital camera (DP22) mounted on a stereomicroscope (Olympus SZX7) with Olympus CellSens Entry v1.18 software. The measuring method of morphometric characters follows that of von Rintelen and Cai (2009). The following abbreviations are used throughout the text: alt (altitude), cl (carapace length), rl (rostral length), and tl (total length). All measurements are in millimeters. Specimens were deposited in the Department of Animal Science, School of Life Science and Engineering, Foshan University (**FU**).

### Molecular data and analysis

Genomic DNA was extracted from abdominal muscle tissue using the Universal Genomic DNA Kit (Beijing, China), following manufacturer instructions. Segments of the mitochondrial cytochrome coxidase I (COI) were amplified by using the primers LCO1490 and HCO2198 (Folmer et al. 1994). PCRs were run in 50 μl volume reactions. COI cycling conditions were: 94 °C for 3 min followed by 35 × (94 °C for 30 s/46±1 °C for 60 s/72 °C for 60 s), with a final elongation step of 5 min at 72 °C. PCR products were forwardly sequenced using primers with an Applied Biosystems 3730 Analyzer (Applied Biosystems, Foster City, CA, USA).

For the phylogenetic analyses, we included species similar in morphology to the new species and also other *Caridina* species that are known to occur in the neighboring areas. A total of 47 nucleotide sequences of 13 *Caridina* and 3 *Neocaridina* species and an outgroup (Table 1) were incorporated and analyzed with MAFFT v7.313 (Katoh and Standley 2013). All the COI sequences were obtained by PCR. The new sequencing results are corrected for 617~bp (COI) for subsequent analysis. All the sequences were aligned with Mega 7.0 (Kumar et al. 2016) software using the muscle alignment module. Genetic distances were calculated using Maximum Composite Likelihood. The best partitioning strategy was selected according to ModelFinder (Kalyaanamoorthy et al. 2017) Bayesian information criterion (BIC) and default parameters. MrBayes v.3.2.6 (Ronquist et al. 2012) was performed using PhyloSuite v1.2.2 (Zhang et al. 2020). Maximum likelihood (ML) was performed using IQ-TREE 1.6.12 (Nguyen et al. 2015). According to the Bayes information criterion (ModelFinder default recommendation), the best model for Bayesian inference (BI) and maximum likelihood method (ML) are GTR+I+G and TIM2+G+I, respectively. Markov chain Monte Carlo (MCMC) analysis was performed with two simultaneous runs starting with random trees to approximate the posterior probabilities of trees. Each run consisted of four chains, with default heating parameters and ran for 4×10^6^ generations, discarding the first 25% as burnin.

**Table 1.** Species used in the molecular analysis, with details on sampling locations and GenBank accession numbers (COI).

Species	Sampling locality	GenBank numbers
*Caridina incolor* sp. nov.	Libo, Guizhou	MW237749–MW237753
*Caridina sinanensis*	Sinan, Guizhou	MT433962–MT433964
*Caridina zhongshanica*	Zhongshan, Guangdong	MN701597–MN701598
*Palaemon modestus*	Dongting lake, Hunan	MK412768–MK412769
*Neocaridina denticulata*	Hulun lake, Inner Mongolia	MW222157–MW222159
*Neocaridina palmata*	Hong Kong, China	MW226891–MW226893
*Caridina mariae*	Nankun Mountarin, Huizhou	MN701601–MN701602
*Caridina serrata*	Dong’ao Island, Zhuhai	MN701599–MN701600
*Caridina tetrazona*	Zhuhai, Guangdong	MN701593–MN701594
*Caridina cantonensis*	Qingyuan, China	MN701589–MN701590
*Caridina* sp. 2	Huanjiang, Guangxi	MW237763–MW237764
*Caridina huananensis*	Yingde, Qingyuan	MN701607–MN701608
*Caridina lanceifrons*	Dongfang, Hainan	MN701605–MN701606
*Caridina zhujiangensis*	Dong’ao Island, Zhuhai	MN701603–MN701604
*Caridina carvernicola*	Mashan, Guangxi	MW237867
*Neocaridina heteropoda*	Guilin, Guangxi	MW221964–MW221966
		MW222154–MW222156
*Caridina* sp. 1	Mashan, Guangxi	MW237861–MW237866

**Table 2.** Pairwise genetic distance among 17 species based on COI (below diagonal) gene.

	**Species**	**1**	**2**	**3**	**4**	**5**	**6**	**7**	**8**	**9**	**10**	**11**	**12**	**13**	**14**	**15**	**16**	**17**
1	*Caridina incolor* sp. nov.																	
2	*Caridina sinanensis*	0.13																
3	*Caridina zhongshanica*	0.17	0.14															
4	*Palaemon modestus*	0.24	0.20	0.19														
5	*Neocaridina denticulata*	0.18	0.14	0.15	0.20													
6	*Neocaridina palmata*	0.18	0.14	0.16	0.21	0.05												
7	*Caridina mariae*	0.17	0.15	0.08	0.21	0.16	0.17											
8	*Caridina serrata*	0.16	0.12	0.11	0.21	0.14	0.16	0.12										
9	*Caridina tetrazona*	0.17	0.14	0.12	0.21	0.17	0.17	0.13	0.09									
10	*Caridina cantonensis*	0.16	0.14	0.01	0.19	0.15	0.16	0.08	0.11	0.12								
11	*Caridina* sp. 2	0.15	0.11	0.14	0.21	0.15	0.15	0.15	0.13	0.14	0.15							
12	*Caridina huananensis*	0.16	0.12	0.14	0.21	0.15	0.17	0.14	0.13	0.14	0.14	0.14						
13	*Caridina lanceifrons*	0.19	0.18	0.18	0.22	0.20	0.18	0.19	0.18	0.18	0.18	0.19	0.19					
14	*Caridina zhujiangensis*	0.18	0.16	0.17	0.20	0.18	0.18	0.19	0.18	0.19	0.17	0.16	0.18	0.17				
15	*Caridina carvernicola*	0.16	0.12	0.14	0.21	0.14	0.15	0.14	0.13	0.13	0.14	0.02	0.13	0.20	0.15			
16	*Neocaridina heteropoda*	0.18	0.14	0.16	0.20	0.13	0.15	0.16	0.15	0.15	0.16	0.16	0.16	0.18	0.20	0.15		
17	*Caridina* sp. 1	0.16	0.11	0.14	0.21	0.15	0.15	0.14	0.14	0.14	0.14	0.05	0.14	0.19	0.15	0.03	0.15	

## Taxonomic account

### Family Atyidae De Haan, 1849

#### Subfamily Atyinae De Haan, 1849

**Genus *Caridina* H. Milne Edwards, 1837**

##### Caridina incolor sp. nov.

Taxon classificationAnimaliaDecapodaAtyidae

41D0F7F5-4180-59B4-BC70-CAA92B439824

http://zoobank.org/FF693EA8-140F-49F5-8B85-A59080737670

Figures 3 , 4 , 5

###### Material examined.

***Holotype***: Adult male (FU, 2018-11-27-01), tl 21.8 mm, cl 5.1 mm, rl 3.0 mm; Yaoshui Cave, Mengtang Village, Wangmeng Township, Daqikong scenic area, Libo County, Guizhou Province, China (25°17'1"N, 107°45'8"E, alt. 520.0 m), November 27, 2018.

***Paratypes***: 1 male (FU, 2018-11-27-02) tl 25.9 mm, cl 6.1 mm, rl 3.1 mm; 16 males (FU, 2018-11-27-03) tl 17.4–25.9 mm, cl 4.2–6.0 mm, rl 2.4–3.4 mm; 10 females (FU, 2018-11-27-04), tl 17.8–25.2 mm, cl 4.5–6.1 mm, rl 2.4–3.4 mm; cl 4.9–6.6 mm, same data as for holotype. ***Paratypes***: 6 males (FU, 2018-11-27-05) tl 18.4–25.1 mm, cl 4.4–5.9 mm, rl 2.5–3.0 mm; 9 females (3 ovigerous) (FU, 2018-11-27-06), tl 17.5–25.0 mm, cl 4.5–6.0 mm, rl 2.4–3.1 mm; cl 5.0–6.3 mm, same data as for holotype. Two samples from (FU, 2018-11-27-05) and three samples from (FU, 2018-11-27-06) were sequenced.

###### Comparative material examined.

*Caridina sinanensis* Xu, Li, Zhang and Guo 2020. Holotype: Adult male (FU, 2019-01-25-01), tl 16.7 mm, cl 4.8 mm, rl 1.5 mm; a cave river at Pengjiaao, Tangtou Town, Sinan County, Guizhou Province, southwestern, China (27°44'10"N, 108°11'58"E, alt. 294.7 m), January 25, 2019. Paratypes: 1 male (FU, 2019-01-25-02) cl 5.4 mm; 1 male (FU, 2019-01-25-03) cl 6.8 mm; 1 male (FU, 2019-01-25-04) cl 4.8 mm; 2 males (FU, 2019-01-25-05), cl 4.2–6.2 mm; 20 females (9 ovigerous) (FU, 2019-01-25-05), cl 4.9–6.6 mm.

###### Diagnosis.

Body and appendages without coloration, translucent. Rostrum slender, slightly elevated at base, reaching to base of 3^rd^ segment of antennular peduncle to end of scaphocerite; straight, slightly sloping downwards, sometimes with tip turned upwards; rostral formula 6–10+11–27/4–15. First pereiopod carpus 0.83–0.91 × as long as chela, 2.3–2.7 × as long as high; chela 2.2–2.5 × as long as broad; fingers 1.1–1.4 × as long as palm. Second pereiopod carpus 1.3–1.4 × as long as chela, 5.4–5.6 × as long as high; chela 2.4–2.6 × as long as broad; fingers 1.6–1.8 × as long as palm. Third pereiopod propodus 3.8–4.0 × as long as dactylus, 13.6–14.4 × breadth, with 8–11 thin spines on the posterior and lateral margins. Fifth pereiopod propodus 4.0–4.7 × as long as dactylus, 17.6–20.5 × breadth, with 17–20 thin spines on the posterior and lateral margins, dactylus terminating in one claw, with 50–55 spinules on flexor margin. Endopod of male subrectangular, slightly wider proximally, length 0.39–0.46 × exopod length, 2.0–2.2 × proximal breadth, ending broadly rounded; inner margin slightly concave, bearing spine-like setae, outer margin slightly convex, proximally 1/3 bearing nearly equal length short spine-like setae, distally 2/3 bearing nearly equal length long spine-like setae, and top bearing nearly equal length stout spine-like setae; appendix interna well developed, arising from distal 1/3 of endopod, beyond the end of endopod, distally with cincinulli. Appendix masculina rod-shaped and gradually tapering into a triangular tip, reaching about 0.48–0.51 × length of endopod, with numerous long spined setae on proximal and distal regions; endopod reaching about 0.76–0.79 × length of exopod; appendix interna well developed, reaching about 0.58–0.78 × length of appendix masculina, with cincinulli distally. Uropodal diaeresis with 10–12 movable spinules. Females carry 10–15 eggs, size of undeveloped eggs (without eyespots) 0.83–0.88 × 1.18–1.26 mm.

**Figure 3.**
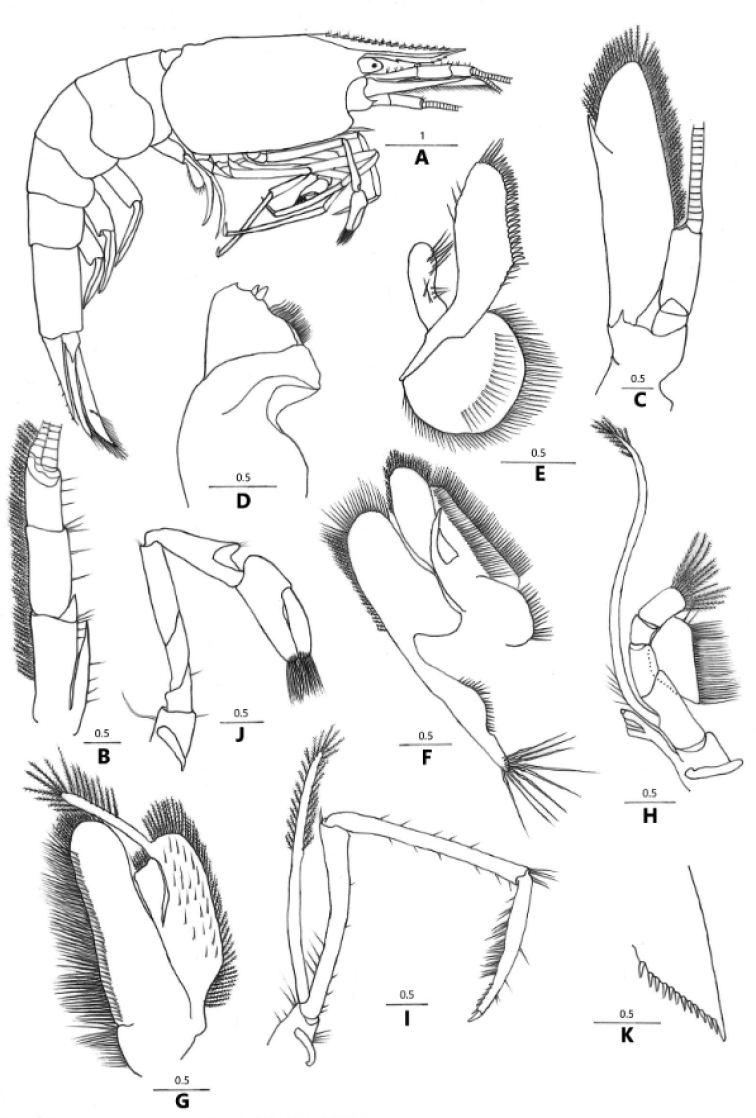
*Caridina incolor* sp. nov. **A** entire animal, lateral view **B** antennule **C** antenna **D** mandible **E** maxillula **F** maxilla **G** first maxilliped **H** second maxilliped **I** third maxilliped **J** first pereiopod **K** diaeresis of uropodal exopod **A** holotype **B–K** paratype (FU, 2018-11-27-02).

###### Description.

***Body*** (Figs 3A, 5C–F): Slender, smooth, colorless, translucent, sub-cylindrical, and medium-sized, males up to 25.9 mm tl, females up to 25.2 mm tl.

**Figure 4.**
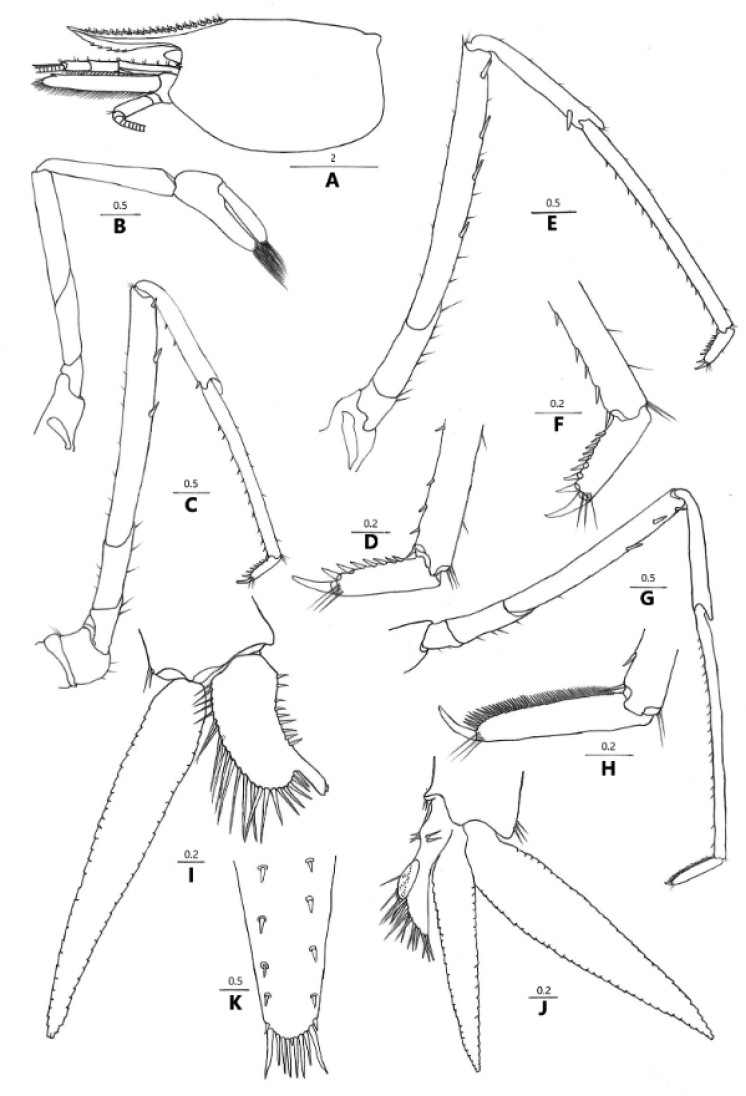
*Caridina incolor* sp. nov. **A** carapace and cephalic appendages, lateral view **B** second pereiopod **C** third pereiopod **D** dactylus of third pereiopod **E** fourth pereiopod **F** dactylus of fourth pereiopod **G** fifth pereiopod **H** dactylus of fifth pereiopod **I** first male pleopod **J** second male pleopod **K** distal margin of telson **A–K** paratype (FU, 2018-11-27-02).

**Figure 5.**
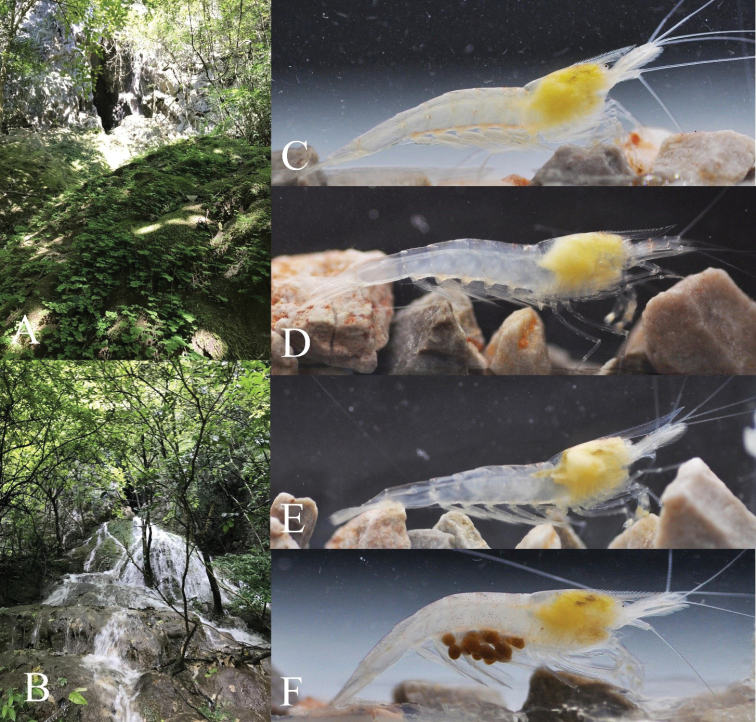
Habitats, variable features, and live coloration of *Caridina incolor* sp. nov. **A** surrounding environment of Yaoshui Cave during the dry season **B** same site during the rainy season **C–E** variable rostrum and eye size in life **F** ovigerous female in life.

***Rostrum*** (Figs 3A, 4A, 5C–F): 0.47–0.77 of cl, slender, slightly elevated at base, reaching to base of 3^rd^ segment of antennular peduncle to end of scaphocerite; straight, slightly sloping downwards (55.8%, N = 43), tip sometimes turned upward (44.2%); armed dorsally with 17–27 teeth, including 6–10 (usually 5–8) on the carapace, with 4–15 (usually 5–8) ventral teeth; lateral carina dividing rostrum into two unequal parts, continuing posteriorly to the orbital margin.

***Eyes*** (Figs 3A, 4A, 5C–F) small, bullet-like, reduced, with a short stalk, lacking facets, cornea pigmentation has great variability in the eye phenotype, with the smaller pigmented cornea (79%, N = 43), or totally pigmentless and blind (21%).

***Carapace*** (Figs 3A, 4A): Smooth, swollen; antennal spine acute, fused with inferior orbital angle; pterygostomial angle angular, produced forward; pterygostomian spine absent.

***Antennule*** (Fig. 3B): Reaching slightly short of scaphocerite; stylocerite long, reaching end of the basal segment, basal segment robust, anterolateral angle with broadly produced sharp projection, reaching 0.25 length of 2^nd^ segment; about 0.72–0.76 × of combined length of 2^nd^ and 3^rd^ segments, 2^nd^ segment as long as 0.76–0.82 × of basal segment, 1.2–1.3 × of 3^rd^ segment; all segments with sub-marginal plumose setae.

***Antenna*** (Fig. 3C): Peduncle about 0.44–0.58 × as long as scaphocerite; scaphocerite 3.1–3.8 × as long as wide, outer margin straight, asetose, ending in a strong sub-apical spine, inner and anterior margins with long plumose setae.

***Mouthparts*** characteristic of the genus. Mandible with well-developed incisor and molar processes; left incisor process with a single short sharp outer tooth, two long stout inner teeth, 7 curving setae followed by a patch of long setae; molar process stout and with triturative surface (Fig. 3D). Maxillula with broadly rounded lower lacinia, with several rows of marginal and submarginal plumose setae; upper lacinia elongate, medial edge straight, with 25–35 strong spinules and simple setae; palp simple, longer than wide, slightly expanded distally, with 4 simple setae at basal part and 6 at distal part (Fig. 3E). Maxilla with well-developed scaphognathite, tapers posteriorly, with a regular row of long plumose setae and short marginal plumose setae continuing down the proximal triangular process distally, furnished with numerous long plumose setae; upper and middle endite with marginal, simple, denticulate setae, with plumose setae distally; lower endite with long simple marginal setae; palp shorter than the cleft of upper endite, wider than distal setose proximally (Fig. 3F). First maxilliped palp broad, with terminal plumose setae; caridean lobe broad, with marginal plumose setae; exopodal flagellum well developed, with marginal plumose setae distally; ultimate and penultimate segments of endopod indistinctly divided; medial and distal margins of an ultimate segment with marginal and sub-marginal rows of simple, denticulate and plumose setae; penultimate segments with marginal long plumose setae (Fig. 3G). The second maxilliped with endopodite ultimate and penultimate antennomeres fused, slightly concave, reflected against basal antennomeres, inner margin of ultimate, penultimate, and basal segments with long setae of various types; exopod flagellum long, slender with marginal plumose setae distally. Podobranchium comb-like (Fig. 3H). Third maxilliped with three-segmented endopod, reaching slightly beyond scaphocerite; penultimate segment 0.89–0.96 × of the basal segment; distal segment 0.75–0.91 × of the penultimate segment, ending in a large claw-like spine surrounded by simple setae, preceded by about 7–9 spines on the distal third of the posterior margin, with a clump of long and short simple, serrate setae proximally; exopod flagellum well developed, about 0.25–0.34 × of the penultimate segment of endopod, distal margin with long plumose setae (Fig. 3I).

***First pereiopod*** (Fig. 3J): Short, reaches the end of eyes; chela length 2.2–2.5 × breadth, 1.1–1.2 × length of the carpus; movable finger length 3.1–3.4 × breadth, 1.1–1.4 × length of the palm, setal brushes well developed; carpus excavated disto-dorsally, length 2.3–2.7 × breadth, about the same length of merus.

***Second pereiopod*** (Fig. 4B): Reaches about the end of 3^rd^ antennular peduncle segment, slenderer and longer than the first pereiopod; chela length 2.4–2.6 × breadth, 0.73–0.77 × length of the carpus; movable finger length 4.5–4.8 × breadth, and 1.6–1.8 × length of the palm, setal brushes well developed; carpus length 5.4–5.6 × breadth, slightly excavated distally, 1.0–1.1 × length of merus.

***Third pereiopod*** (Fig. 4C, D): Reaches beyond the end of scaphocerite; dactylus length 4.4–4.7 × breadth, ending in prominent claw-like spine surrounded by simple setae, behind which bears 6–7 spines; propodus length 3.8–4.0 × of the dactylus, bearing 8–11 spinules on posterior margin, 13.6–14.4 × breadth; carpus length 0.65–0.72 × of the propodus; merus length 1.9–2.1 × of the carpus, with about 3 strong spines on the posterior margin.

***Fourth pereiopod*** (Fig. 4E, F): Reaches end of 3^rd^ segment of antennular peduncle; dactylus length 4.2–5.0 × breadth, ending in prominent claw-like spine surrounded by simple setae, behind which bears 7–8 spines; propodus length 3.8–4.6 × of the dactylus, bearing 12–15 spinules on posterior margin, 14.6–17.4 × breadth; carpus length 0.58–0.73 × of the propodus; merus length 1.9× of the carpus, with about 3 strong spines on the posterior margin.

***Fifth pereiopod*** (Fig. 4G, H): Reaches end of 3^rd^ segment of antennular peduncle; dactylus length 4.2–5.6 × breadth, ending in prominent claw-like spine surrounded by simple setae, behind which bears comb-like row 50–55 spines; propodus length 4.0–4.7 × of the dactylus, bearing 17–20 spinules on posterior margin, 17.6–20.5 × breadth; carpus length 0.55–0.60 × of the propodus; merus length 1.5–1.6 × of the carpus, with about 3 strong spines on the posterior margin.

First four pereiopods with epipod. Branchial formula typical for the genus.

***First pleopod*** (Fig. 4I): Endopod of male subrectangular, slightly wider proximally, length 0.39–0.46 × exopod length, 2.0–2.2 × proximal breadth, ending broadly rounded; inner margin slightly concave, bearing spine-like setae, outer margin slightly convex, proximally 1/3 bearing nearly equal length short spine-like setae, distally 2/3 bearing nearly equal length long spine-like setae, and top bearing nearly equal length stout spine-like setae; appendix interna well developed, arising from distal 1/3 of endopod, reaching beyond the end of endopod, with cincinulli distally.

***Second pleopod*** (Fig. 4J): Appendix masculina rod-shaped and gradually tapering into a triangular tip, reaching about 0.48–0.51 × length of endopod, with numerous long spine setae on proximal and distal margins; endopod reaching about 0.76–0.79 × length of exopod; appendix interna well developed, reaching about 0.58–0.78 × length of appendix masculina, with cincinulli distally.

***Telson*** (Fig. 4K): 0.40–0.55 × of cl, shorter than sixth abdominal segment, 0.67–0.96 × length of the sixth abdominal segment, posteriorly tapering, with median projection, dorsal surface with 5 pairs of stout movable spine setae including the pair at posterolateral angles; posterior margin with 4 pairs of intermedial strong spiniform setae, sublateral pair shorter than lateral and inner pairs. Exopodite of the uropod bears a series of 10–12 movable spinules along the diaresis, the last one shorter than the lateral process (Fig. 3K).

Females carry 10–15 eggs, size of undeveloped eggs (without eyespots) 0.83–0.88 × 1.18–1.26 mm.

***Coloration*** (Fig. 5C–F): Body and appendages are colorless and translucent; vestigial pigment present at the center of the cornea or without pigment; internal organs (gonads and hepatopancreas) are yellow; eggs in females brown.

###### Etymology.

*Caridina incolor* sp. nov. is named after the colorless and transparent body color.

###### Remarks.

*Caridina incolor* sp. nov. might be more closely related to the epigean species than to its supposed cave congeners. It is morphologically similar to *C. guiyangensis* Liang, 2002, from Guiyang, Guizhou Province in the long rostrum and indentation, the shape of endopod of the male first pleopod and appendix masculina. Although no molecular comparison with *C. guiyangensis* could be accomplished, *C. incolor* sp. nov. can easily be distinguished from the latter by the reduced eyes, colorless body and appendages (versus developed eyes and pigmentation in *C. guiyangensis*); the long stylocerite, reaching to the end of the antennule basal segment (versus reaching 0.85 × of basal segment in *C. guiyangensis*); the long penultimate segment of the 3^rd^ maxilliped, which is distinctly longer than the basal segment and distal segment (versus penultimate segment as long as basal segment and distinctly shorter than distal segment in *C. guiyangensis*); absence of a projection on the base of the inner margin of male first pleopod endopod (vs. with projection in *C. guiyangensis*); and relatively large eggs, size of undeveloped eggs 0.83–0.88 × 1.18–1.26 mm (versus 0.63–0.75 × 1.05–1.13 in *C. guiyangensis*). In comparison to other cave species within *Caridina*, *C. sinanensis* Xu, Li, Zhang & Guo, 2020, is most similar in sharing the long 6^th^ abdominal segment, and the variably pigmented cornea. However, the new species differs from the latter by possessing a relatively long and slender rostrum which reaches beyond the end of the 3^rd^ antennular peduncle segment (versus stouter, reaching to the end of the 2^nd^ segment in *C. sinanensis*); the long stylocerite, reaching to the end of the basal segment of the antennule (versus reaching 0.75–0.88 × of basal segment in *C. sinanensis*); and completely different shape of the endopod of the 1^st^ pleopod and appendix masculina of the 2^nd^ pleopod in males (Fig. 4I, J, versus Fig. 4H, J, K in Xu et al. 2020).). There are another six atyids, *Caridina acuta*, *C. caverna*, *C. demenica*, *C. jiangkou*, *C. sinanensis*, and *Neocaridina brevidactyla*, that have been reported from nearby caves from Guizhou Province. *Caridina incolor* sp. nov. differs from *N. brevidactyla* in the completely different shape of the endopod of 1^st^ pleopod and appendix masculina of the 2^nd^ pleopod in males; lack of a hook-like projection on the posterior part of coxa of the 2^nd^ pereiopod and a pterygostomian spine. *C. incolor* sp. nov. differs from all other cave species in having a long rostrum and stylocerite (with the longest rostrum and stylocerite amongst all known seven cave atyids); slender sixth abdominal segment which is distinctly longer than the telson (only *C. sinanensis* has a slender sixth abdominal segment that is slightly longer than the telson, other cave species have a stout sixth abdominal segment which is distinctly shorter than the telson); and an appendix masculina that is unique in shape and gradually tapers into a triangular tip. These taxa can be separated from each other by morphological differences as discussed in Xu et al. (2020).

###### Molecular phylogenetic results.

We analyzed 47 COI sequences in total. Five specimens of *Caridina incolor* sp. nov. were used in the molecular phylogenetic analysis shown in Figure 2. Specimens assigned to *Caridina incolor* sp. nov. formed a clade distinct from other species. *Caridina incolor* sp. nov. is well distinguished from the other 16 atyids with a sequence divergence of 13.7% – 24.5% (COI). The sequence divergence between *Caridina incolor* sp. nov. and *C. sinanensis* is the closest. The topology of the Bayesian (BI) trees and the ML tree are basically similar. Phylogenetic trees revealed the relationship between *Caridina incolor* sp. nov. and 16 other species of atyids, with the posterior probability and bootstrap values from the BI and ML analysis shown in Figures 2. According to Hebert, Ratnasingham and de Waard 2003, the genetic distances supports *Caridina incolor* sp. nov. as a new species.

###### Conservation.

Threats to cave shrimp are of concern due to the uniqueness of the habitat and increasing anthropogenic activities. Based on the information available, the Yaoshui Cave and its faunas are potentially at risk from excessive levels of external disturbance. Daqikong scenic area is famous for its marvelous primeval forests, steep canyons, spectacular caves, and underground rivers. Over the years, tourism to the region has improved the welfare of local residents and has become a major industry in this area. Moreover, new recreational trails and amusement facilities have been built in the scenic area. It is almost inevitable that these new projects will put great pressure and impact on caves and their faunas. In addition, land development and agriculture lead to habitat degradation and groundwater pollution, which also has a negative impact on the survival of this species.

So far, no freshwater shrimps are protected by the national legislation. The Announcement of the Ministry of Agriculture and Rural Areas of China has failed to categorize the strictly endemic cave species as Endangered (CITES Appendix aquatic wild species of China, no. 69, 2018). *Caridina incolor* sp. nov. is new to science and the conservation status remains unassessed. Using the criteria provided by the IUCN (2019) Red List Categories and Criteria (version 14) (IUCN (2019)), the new species should be considered as a critically endangered (CR) species on account for its exceptional rarity, restricted distribution, and exposure to serious anthropogenic impacts.

The Yaoshui Cave, which is home to two unique and range-restricted species (atyid shrimp, *Caridina incolor* sp. nov., and loach fish, *Oreonectes daqikongensis*) is biologically significant without question. These strictly-adapted cave species must be considered as important units for conservation, and devising an effective conservation strategy is clearly an urgent priority. It has also become obvious that there is a need to collect more baseline data, such as the exact population size, structure, natality, and mortality rates. Regular monitoring may be necessary to ensure populations are sustained in the face of further anthropogenic disturbances. Equally important, a captive breeding program of these cave species should be developed. In addition, we propose that non-invasive and non-destructive projects, such as eco-tourism, should be promoted. Last but not least, we also appeal to local farmers to lower the usage of agricultural pesticides, herbicides, and fertilizers to help reduce the amount of hazardous chemicals that are leeched into the groundwater.

## Supplementary Material

XML Treatment for Caridina incolor
